# Gene expression analysis reveals genes related to heavy metals and produced water exposure in Synechococcus elongatus

**DOI:** 10.1007/s10123-025-00715-x

**Published:** 2025-09-22

**Authors:** Alaa Hassanien, Nisar Ahmed, Borbala Misfud, Hareb M. Al-Jabri, Sara Al-Marri, Tasneem Dalgamouni, Maryam Al-Merekhi, Kira Schipper, Imen Saadaoui, Suhur Saeed, Mustapha Aouida

**Affiliations:** 1 Algal Technologies Program, Center for Sustainable Development, College of Arts and Sciences, , Qatar University, P.O. Box 2713, Doha, Qatar; 2 Biological and Environmental Sciences Department, College of Arts and Sciences, Qatar University, P.O. Box 2713, Doha, Qatar; 3 Division of Genomic and Precise Medicine, College of Health and Life Sciences, Hamad Bin Khalifa University, Education City, Qatar Foundation, Doha, P.O.Box: 34110, Qatar; 4 ExxonMobil Research Qatar (EMRQ), , P.O. Box 22500, Doha, Qatar; 5 Division of Biological and Biomedical Sciences, College of Health and Life Sciences, Bin Khalifa University, Education City, Qatar Foundation, P.O.Box: 34110, Doha, Qatar; 6 Institute for Molecular Bioscience, The University of Queensland, 306 Carmody Road, Brisbane, QLD 4072 Australia

**Keywords:** Produced water, Bioremediation, Transcriptomics, Wastewater, Cyanobacteria

## Abstract

Produced water (PW), a major by-product of the petrochemical industry, contains a complex mixture of contaminants that limit its reuse and pose environmental risks if discharged untreated. Numerous treatment technologies have been developed to remediate this water, with bioremediation standing out as one of the most promising novel approaches. One such bioremediation method is through the application of cyanobacteria, which are able to remove pollutants such as heavy metals from produced water, although the mechanism by which the pollutants are removed is still unknown. In this study, a well-characterized cyanobacterium, *Synechococcus elongatus*, was used as a model organism to establish a proof of concept for identifying genes responsive to PW exposure and heavy metal stress. RNA sequencing was performed to analyze transcriptomic changes in *S. elongatus* grown in BG-11 (control) and exposed to 3 mg/mL of iron (heavy metal (HM)) or 25% v/v PW in BG-11. Differential expression analysis revealed that 11 and 67 genes were ≥ fivefold upregulated, and 337 and 27 genes were ≥ fivefold downregulated under HM and PW exposure, respectively, compared to the control. Among the over-expressed genes, the plasma membrane transporter, nitrate ABC transporter permease, was identified, suggesting its important role in the bioremediation process of heavy metals from wastewater. These findings provide foundational insights into stress-responsive gene networks in cyanobacteria and inform future bioengineering strategies for enhancing bioremediation capabilities in *S. elongatus* and related strains.

**Supplementary Information:**

The online version contains supplementary material available at 10.1007/s10123-025-00715-x.

## Introduction

The growth of the human population and the increasing demand for resources such as water, food, and energy have led to the rapid expansion of industries, resulting in the unavoidable co-production of waste streams. Within petroleum industries, one such waste-stream is produced water, a naturally occurring water that is co-extracted along with oil and gas during the extraction processes. It often contains a mixture of soluble and non-soluble oil/organics, suspended solids, dissolved solids, and various chemicals used in the production process, as well as heavy metals such as iron, copper, manganese, cadmium, lead, nickel, chromium, and mercury (Goswami et al. 2017, 2018; Abdelhamid et al. 2024). These heavy metals can pose significant environmental and health risks if not properly managed and treated (Molaei et al. 2020; Kamani et al. 2023).

Several methods, either physical, chemical, and biological, have been developed and implemented to remove such pollutants from contaminated water. Physical and chemical methods include chemical precipitation, ion exchange, adsorption, reverse osmosis, electrodialysis, ultrafiltration, nanofiltration, coagulation, flocculation, and floatation. Although such methods can be effective, often they are costly and energy intensive or release secondary pollutants (Crini and Lichtfouse 2019; Kato and Kansha 2024). The future of produced water treatment therefore requires strategies that can integrate current technologies with innovative strategies to increase sustainability, decrease costs, and use produced water as a resource rather than a waste.

Biological treatment, particularly bioremediation using microalgae, is considered a promising option due to its sustainability and efficiency for metal remediation (Rajasulochana and Preethy 2016). Microalgae have gained interest as an effective wastewater treatment process, capable of removing major contaminants such as nitrogen, phosphorus, heavy metals, and hydrocarbons (Hassanien et al. 2023). Cyanobacteria, in particular, have been extensively used for wastewater treatment and purification, utilizing existing pollutants, either naturally occurring or xenobiotic, and CO_2_ to support growth and produce valuable biological products, biomaterials and bioenergy (Yadav et al. 2020); Li et al. 2021; Abdelfattah et al. 2023). They poses the ability to adapt to adverse environments and synthesize metal-binding proteins (metallothioneins) that bind to inorganic pollutants like heavy metals, via cysteinyl thiolate bridges to cysteine ligands (Kalita and Baruah 2023).

Enhancing bioremediation efficiency, as well as microalgal growth rates, under the presence of such relevant contaminants is of great interest to many industries to further optimize the process. One novel approach to improve algal capabilities and phytoremediation of wastewater is through genetic modification. Genetic modification in microalgae is a promising technology to increase yields and overcome high production costs (Hassanien et al. 2023). Recent advances in genetic engineering, particularly the progressive development of powerful and innovative gene editing tools for microalgal and cyanobacterial metabolic pathways, have opened new opportunities for industrial development and biotechnological application (Grama et al. 2022).

To apply genetic engineering tools, such as the CRISPR-Cas9 system, to enhance the produced water bioremediation process by cyanobacteria and microalgae, it is essential to understand the molecular changes that occur in response to environmental stressors like heavy metals or wastewater. Therefore, in this study, high-throughput RNA sequencing was utilized to investigate the transcriptome profile changes in *Synechococcus elongatus*, a model cyanobacterium, in response to exposure to produced water as well as elevated levels of iron, one of the water’s main contaminants. Understanding the molecular changes by investigating gene expression patterns, including upregulation and downregulation in response to these environmental stressors, can pave the way towards developing more efficient and targeted bioremediation strategies. This knowledge will facilitate the genetic engineering of *Synechococcus elongatus* and other microalgae to enhance their capacity for detoxifying contaminated environments, ultimately contributing to more sustainable and effective wastewater treatment solutions.

## Materials and methods

### Synechococcuselongatus growth assay

*Synechococcus elongatus* UTEX 2973 was obtained from the UTEX algae culture collection (www.utex.org). Stock cultures were maintained in BG-11 media, in an illuminated shaker incubator (Innova 44R, New Brunswick Scientific, USA), 150 rpm, 30 °C, and 70 µmol·m^−2^ s^−1^ Light intensity with 12:12-h light:dark cycles. Once sufficient densities were reached, biomass was used to inoculate flasks (working volume 60 mL) with a initial OD_730_ of 0.3, for 4 conditions: (1) BG-11 (control), (2) BG-11 with 3.0 mg·L^−1^ ammonium ferric citrate, (3) BG-11 with 5% v/v produced water, and (4) BG-11 with 25% v/v produced water. Produced water was not pre-treated to maintain original composition (Supplementary Table S1). Flasks were inoculated in the shaker incubator (150 RPM) at 30 °C and 70 µmol·m^−2^ s^−1^light intensities with 12:12-h light:dark cycles. Optical densities at 730 nm were followed daily over the course of 11 days, and all experiments were performed in triplicate (*n* = 3).

### Synechococcus elongatus gene expression cultivation

Based on the outcomes of 2.2, new cultures of *S. elongatus* were inoculated for gene expression analysis comparisons between BG-11 with 3.0 mg L^−1^ ammonium ferric citrate (HM), BG-11 with 25% v/v produced water (PW), and BG-11 media (control). Produced water was pretreated with UV Light for 1 h prior to inoculation to minimize existing bacterial contamination. All experimental flasks (180-mL working volume) were started with an initial OD_730_ of 0.5, obtained by centrifuging equivalent volumes of *S. elongatus* stock culture at 1500 rpm for 20 min, and resuspending the pellet in the different media conditions. Flasks were incubated under the same conditions as above, and all treatments (including control) were performed in duplicate (*n* = 2). Samples of 15 ml were collected at 0, 4, 8, 16, 24, 48, and 68 h after inoculation, and stored immediately at − 80 °C prior to RNA extraction.

### RNA extraction

RNA extraction from treated and untreated *S. elongatus* was performed using RNeasy Plant Mini Kit (cat no. 74904, Qiagen) according to the manufacturer’s instructions and with the following modifications. Frozen *S. elongatus* samples (stored in − 80 °C) were centrifuged (Eppendorf centrifuge 5810R) at 6000 rpm for 10 min, the pellet was resuspended in 1 mL of RLC lysis buffer, and the mixture was loaded into the supplied 1.5-mL screw cap tubes, with ice-cold zirconia beads. After incubation for 15 min at room temperature, the samples were disrupted (vortex adapter, Grant-bio, Multi-Vortexer V-32) for 10 min at maximum speed, with the tube caps towards the center. The suspension was centrifuged for 10 min at 12,000 rpm at room temperature (Eppendorf, 5424) to separate the aqueous phase, containing the RNA, from the organic phase and samples were then proceeded following steps 2–10 of the Qiagen Rneasy Plant Mini Kit.

### RNA sequencing

RNA sequencing (RNA-seq) of total RNA was conducted at Weill Cornell Medicine – Qatar (WCM-Q) Genomics Core Facility. Following RNA extraction, 400 ng of high integrity total RNA was used to generate strand-specific 300–400-bp libraries using NEXTflex Rapid Directional RNA-Seq Kit (Bioo-Scientific, USA Catalog #NOVA-5138–07) according to the manufacturer’s protocol. Library quality and quantity were analyzed with the Bioanalyzer 2100 (Agilent, USA) on a High Sensitivity DNA chip. The Libraries were then pooled in equimolar ratios and paired end 150 bp sequenced on Illumina NextSeq 550.

### RNA-seq data analysis

Raw RNA-seq reads underwent quality control (QC) using FastQC, and adaptors at both ends were trimmed using TrimGalore2. Kallisto3 (Near-optimal RNA-Seq quantification) was employed to align the trimmed reads to the coding sequences (CDS) of *S. elongatus* UTEX 2973 downloaded from the National Center for Biotechnology Information (NCBI) and to quantify transcript abundances. The resulting transcripts per million (TPM) counts were imported into R (version 4.2.0, https://www.r-project.org) for downstream analysis. The TPM counts were normalized using the edgeR4 package, and genes were identified as upregulated (> 1 log2 fold change) or downregulated (< − 1 log2 fold change) based on the comparison (Ahmed et al., 2024). Gene expression change profiles were clustered using *k*-means clustering (*k* = 10) and the resulting clusters were visualized using (Raivo Kolde 2010) and ggplot2 (Wickham 2016). Venn diagrams were generated using the ggvenn R package to compare the overlap of upregulated and downregulated genes between the 24- and 48-h produced water and heavy metal samples.

## Results

**Fig. 1:**
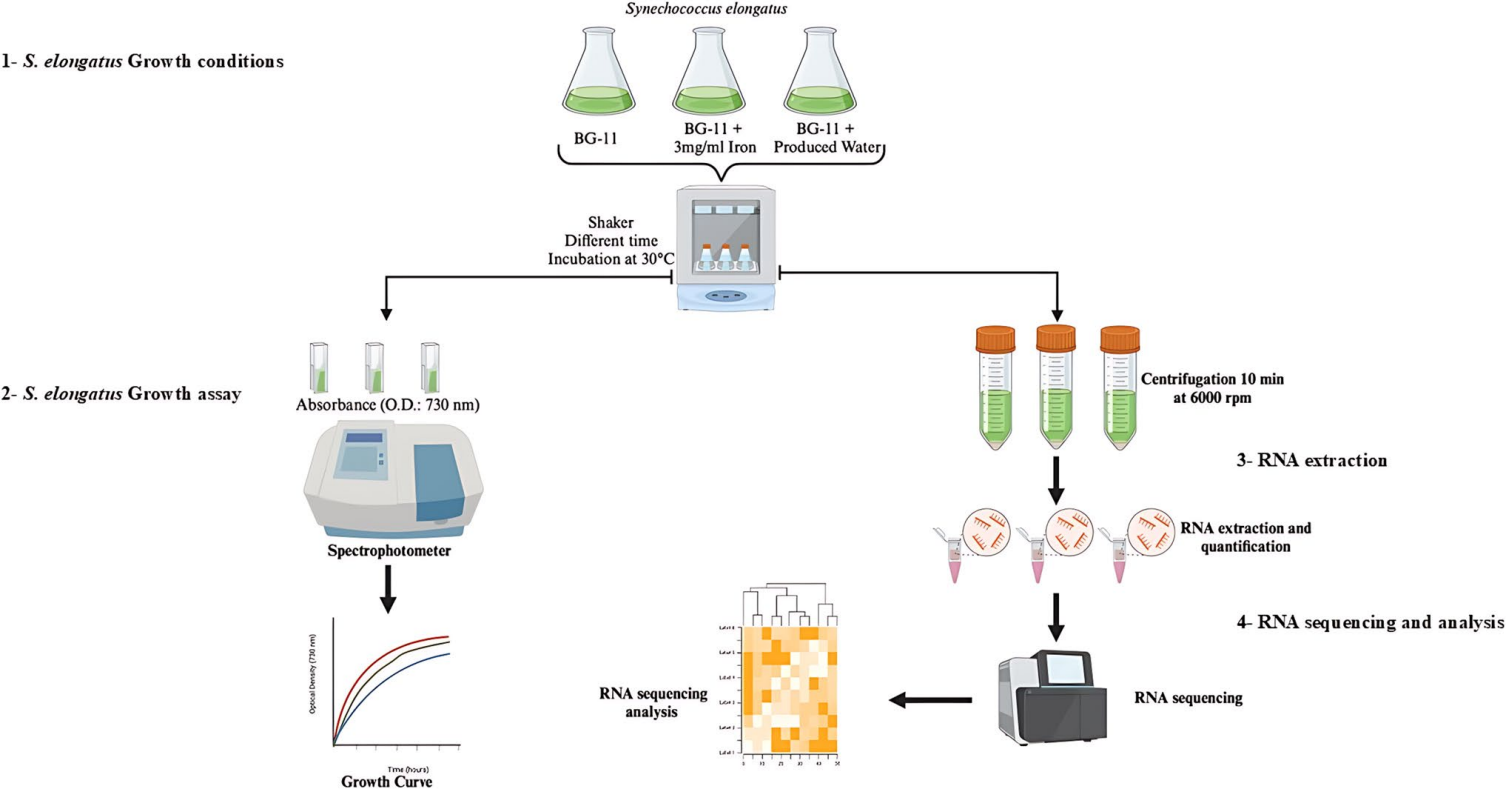
Workflow used in this study to identify genes related to heavy metals and produced water exposure in *Synechococcus elongatus*. The main steps used (1) *S. elongatus* growth conditions; (2) *S. elongatus* growth assay; (3) *S. elongatus* RNA extraction; and (4) *S. elongatus* RNA sequencing and analysis

Produced water treatment is necessary to prevent environmental contamination, whilst sustaining industrial growth to provide for our ever-growing society. Using microalgae for bioremediation has potential, but underlying genetic mechanisms need to be understood to further improve the process for enhanced feasibility. In this study, responses of growth and transcriptome profiles to various conditions were investigated through the workflow as described in Fig. 1.

### Effect of heavy metals and produced water on the growth of S. elongatus

**Fig. 2:**
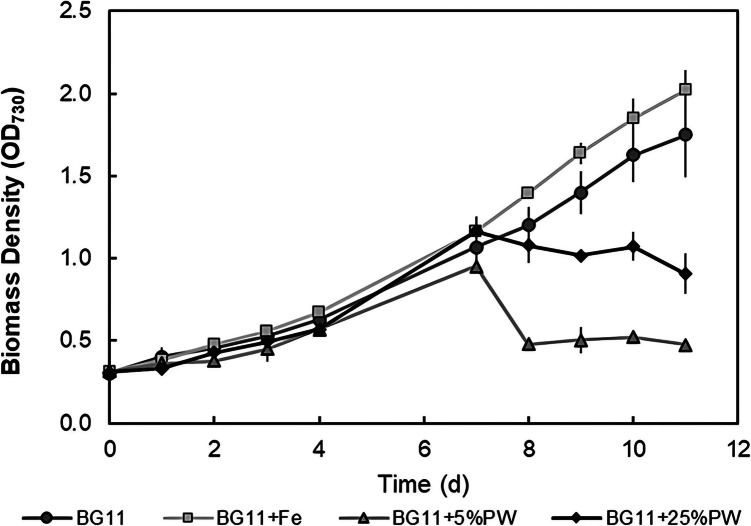
Growth curves of *S. elongatus* under in BG-11 media, BG-11 media with additional iron (BG-11 + Fe), BG-11 media with 5% produced water (BG-11 + 5%PW), and BG-11 media with 25% produced water (BG-11 + 25%PW). Values are mean ± stdev (*n* = 3)

**Fig. 3:**
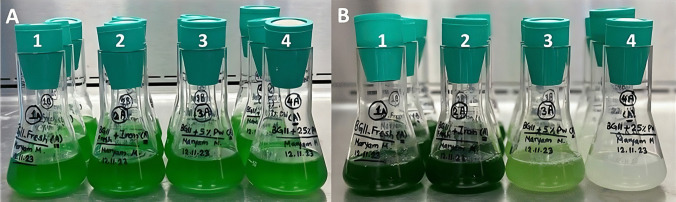
Growth comparison of *S. elongatus* under various media conditions: (1) BG-11 media, (2) BG-11 media with additional iron (BG-11 + Fe), (3) BG-11 media with 5% produced water (BG-11 + 5%PW), and (4) BG-11 media with 25% produced water (BG-11 + 25% PW). Where **A** is the initial culture at time 0 and **B** is at time 11 (days)

The effect of iron (Fe), 5% and 25% (v/v) of produced water (PW) on the growth of *S. elongatus* was evaluated and compared to BG-11 (control) cultures (Fig. 2). Both control and Fe cultures showed similar growth curves, with no significant difference between the two conditions. Although initially the PW cultures showed similar growth curves as compared to the control and Fe cultures, a decline was observed after 7 days (Fig. 2). This was accompanied by a shift in color, from green to white, most prominently for the 25% PW condition (Fig. 3). The decline in algae biomass growth can be most likely attributed to the effect of produced water contaminants. However, it should be noted that the culture with higher produced water content (25%) had higher end optical densities compared to the 5% condition. It was hypothesized that, as OD_730_ measurements are specific to turbidity in general (thus considering bacterial growth as well), high bacterial presence could be responsible for the elevated optical density with higher PW concentrations. This was further suggested by visual observation, by the increased loss of chlorophyll of the culture (Fig. 3), as well as through microscopic observation (data not shown). Bacterial growth in the samples with produced water is not unexpected, as bacteria are already naturally present in the produced water, and the elevated levels of organic carbon originating from the produced water can significantly enhance bacterial growth (Mira et al. 2022).

### Gene expression in S. elongatus under heavy metal and produced water stress

**Fig. 4:**
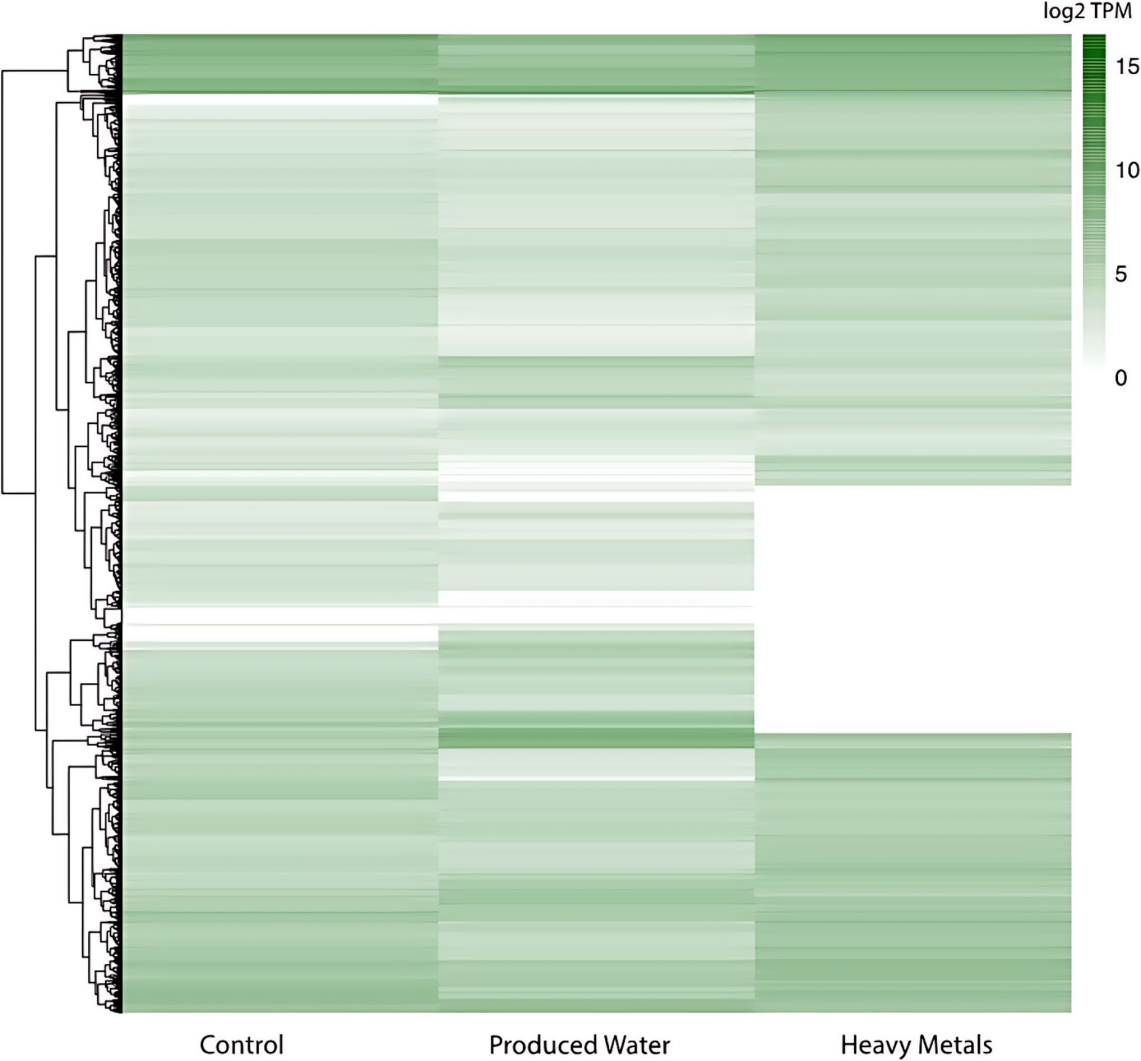
Heatmap of log2-transformed TPM Values in transcriptomic analysis – the log2-transformed values of transcript per million (TPM) for each transcript in control, produced water-, and heavy metal-treated samples at 16 h were hierarchically clustered. Each row within the heatmap corresponds to an individual transcript. The color intensity corresponds to the transcripts’ expression abundance within a given sample; darker shades signify high

Gene expression levels of *S. elongatus* were compared across three different experimental conditions: BG-11 media (control), BG-11 media supplemented with iron (heavy metals), and the most prominent heavy metal in the tested produced water (PW) (Fig. 4). The two treatments had distinct and widespread effects on gene expression. Notably, the repression of several transcripts in response to the treatments was observed, particularly in the HM condition, where a high number of transcripts showed low transcript per million (TPM) reads as compared to the control and PW conditions (Fig. 4 and Supplementary Figure S1). These findings provide insights into the overall gene expression patterns, indicating that *S. elongatus* responds differently to varying growth environments.

To investigate whether there is different set of genes expressing in response to different treatments, differential gene expression analysis was performed by comparing HM versus control, and PW versus control, at different time intervals (0, 4, 8, 16, 24, and 48 h of exposure). This temporal analysis revealed comprehensive dynamics of gene expression, quantifying genes that were either upregulated or downregulated compared to a baseline condition. Additionally, the comparisons showed varying numbers of dysregulated genes across the different conditions. For instance, considering absolute log_2_ fold change (FC), at the 16-h time point within the PW condition, 719 genes were upregulated, and 671 genes were downregulated, while within the HM condition, 699 genes were upregulated, and 766 were downregulated (Supplementary Table S2).

**Fig. 5:**
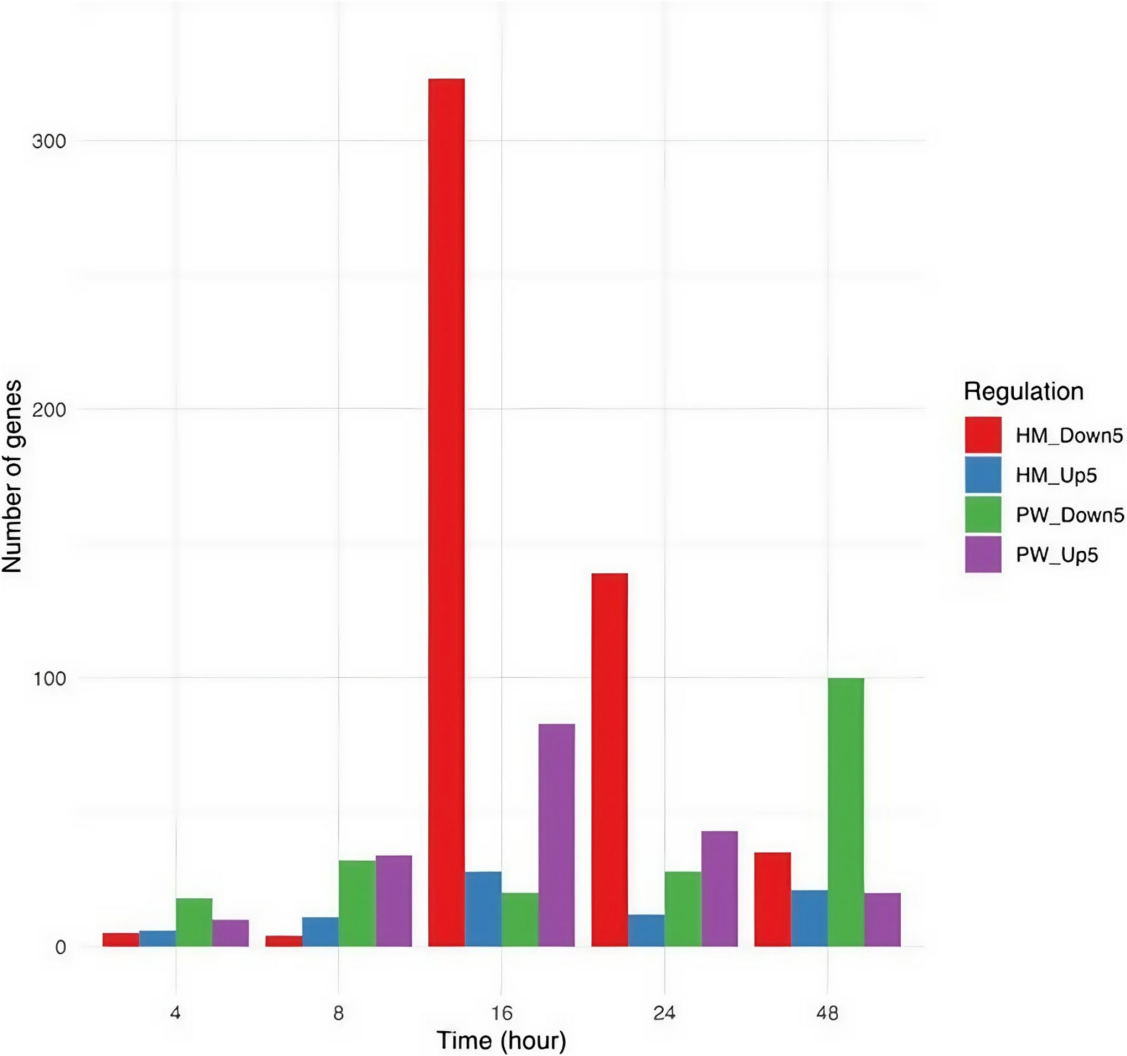
Time course experiments to identify the numbers of genes that are upregulated or downregulated in *S. elongatus* exposed to HM and PW. Number of genes that fivefold over-expressed or down-expressed when *S. elongatus were* exposed for 16 h to 3 mg/mL of HM and 25% PW when compared to the BG-11 control

Furthermore, when investigating the top dysregulated genes across the different conditions (genes with absolute log_2_FC > 5), it was found that the genes at the 16-h time point exhibited a higher level of dysregulated genes compared to other time points across both conditions. For example, for the PW condition, 83 genes were upregulated, 20 genes were downregulated, at the 16-h time point, and within the HM condition, 28 genes were upregulated, and 323 were downregulated (Table 1 and Fig. 5). Consequently, the 16-h time-point data were selected for further downstream analysis.

**Table 1:** List of genes that are dysregulated considering absolute logFC 5 in HM and PW at different time points

Time (h)	PW (upregulated)	PW (downregulated)	HM (upregulated)	HM (downregulated)
4	10	18	6	5
8	34	32	11	4
16	83	20	28	323
24	43	28	12	139
48	20	100	21	35

### Downstream analysis reveals stress-specific and shared gene clusters

**Fig. 6:**
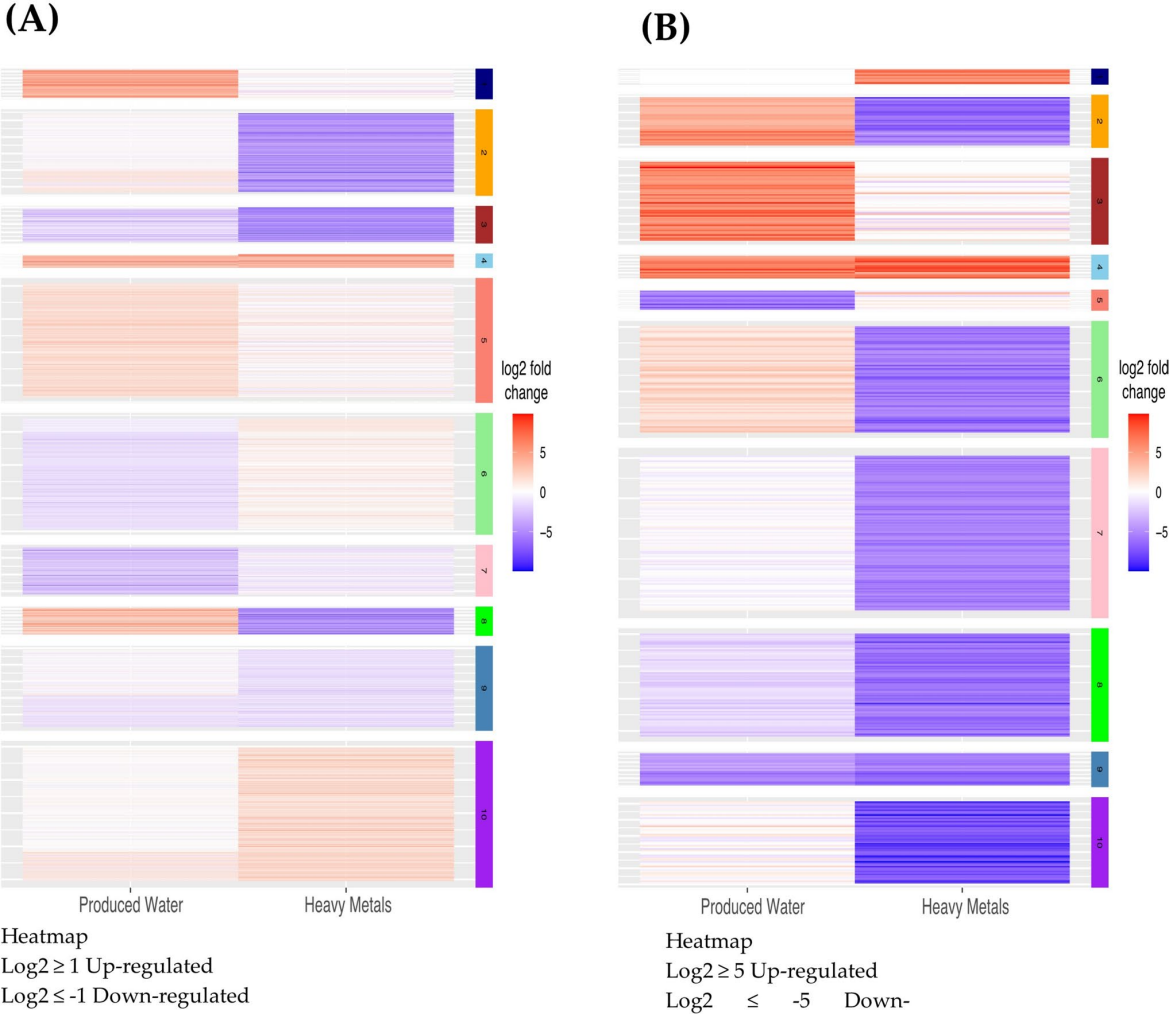
Clustering analysis of gene expression changes in response to heavy metals and produced water treatment—**A** and **B** *k*-means clustering analysis of gene expression changes (absolute log2-fold change threshold > 1 (**A**) and > 5 (**B**) at 16 h of exposure. Each line represents a gene, and each column represents a comparison between the control and treatment groups. On the left side, the comparison is between heavy metals and control, while on the right side, it is between produced water and control. Genes are color-coded, with red indicating upregulated genes and blue indicating downregulated genes

To identify distinct expression patterns between the experimental conditions at 16-h time point, a *k*-means clustering analysis was performed, grouping genes into *k* clusters based on their expression profiles. The clustering analysis revealed distinct expression patterns across the conditions. These patterns were visualized using heatmaps, which highlighted the difference in gene expression in different conditions and identified clusters of co-regulated genes. Notably, it was found that at the 16-h time point very interesting, where we can clearly observe those clusters that indicate key differences between those where BG-11 supplemented with HM and PW. For instance, clusters 2 and 8 with log_2_FC ≤  − 1 (Fig. 6A) and clusters 2, 6, and 10 with log_2_FC ≤  − 5 (Fig. 6B) were downregulated, while clusters 6 and 10 with log_2_FC ≥ 1 (Fig. 6A) and cluster 1 with log_2_FC ≥ 5 (Fig. 6B) were upregulated in HM in comparison to the control. Similarly, cluster 6 with log_2_FC ≤  − 1 (Fig. 6A) and cluster 5 with log_2_FC ≤  − 5 (Fig. 6B) were downregulated, while clusters 1 and 8 with log_2_FC ≥ 1 (Fig. 6A) and clusters 2, 3, and 6 with log_2_FC ≥ 5 (Fig. 6B) were upregulated in PW in comparison to the control. In addition, it was found that cluster 4 with log_2_FC ≥ 1 (Fig. 6A) and cluster 4 with log_2_FC ≥ 5 (Fig. 6B) were upregulated both in HM and PW, whereas cluster 3 with log_2_FC ≤  − 1 (Fig. 6A) and cluster 9 with log_2_FC ≤  − 5 (Fig. 6B) were found downregulated both in HM and PW.

**Fig. 7:**
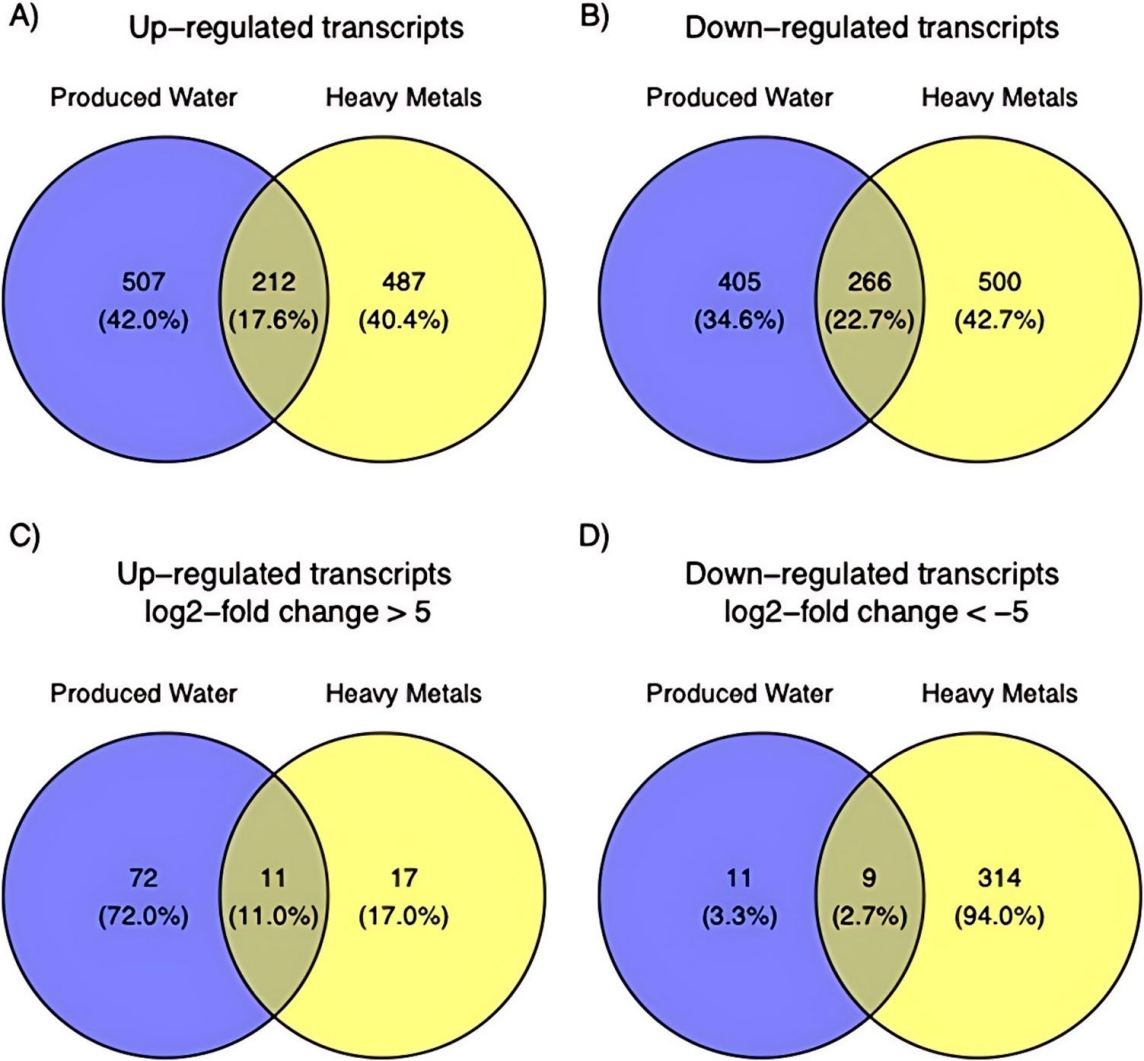
Comparative analysis of gene expression changes in response to produced water and heavy metal treatments—**A** Venn diagram illustrating the overlap of upregulated genes, each exhibiting a log2-fold change greater than 1, in response to both the produced water and heavy metal treatments after 16 h of exposure. **B** The intersection between downregulated genes, each displaying a log2fold change less than − 1, following exposure to the produced water and heavy metal treatments. **C** Similar to **A**, overlap of highly upregulated genes, defined by a log2-fold change greater than 5. **D** Similar to B, overlap of highly downregulated genes, characterized by a log2fold change less than − 5

**Fig. 8:**
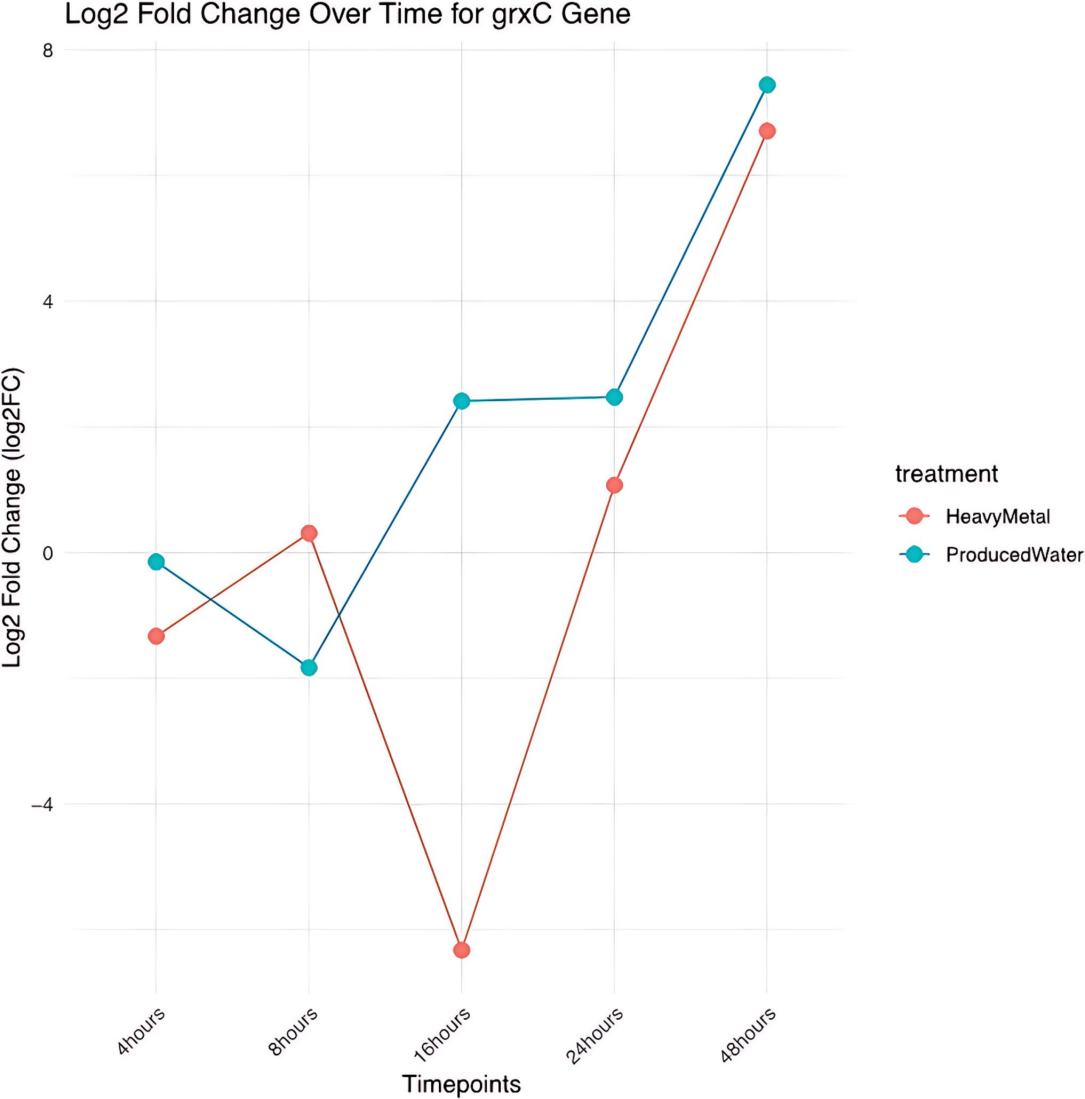
Temporal log2 fold change of gene expression in HM and PW conditions. The dot plot depicts the log2 fold change (log2FC) in gene expression of grxC at different time points for the heavy metal (HM) and produced water (PW) treatment conditions

We further aimed to investigate those clusters that were found to be common between HM and PW at 16-h treatment. We found 162 genes with log_2_FC ≥ 1 (Fig. 7A) and 19 genes with log_2_FC ≥ 5 (Fig. 7C) that were upregulated and shared between PW and HM. In contrast, among downregulated and shared genes between PW and HM, we found 280 genes with log_2_FC ≤ −1 (Fig. 7B) and 42 genes with log_2_FC ≤  − 5 (Fig. 7D). Among the common 19 upregulated genes in both HM and PW, the top 6 genes encode proteins, such as DUF2237 domain-containing protein, exodeoxyribonuclease VII small subunit, DUF1818 family protein, small multi-drug export protein, DUF4160 domain-containing protein, and DUF2281 domain-containing protein (Supplementary Table S3). Whereas the top 6 genes from common 42 downregulated genes in both HM and PW encode proteins, such as circadian clock protein KaiB, PAAR domain-containing protein, J domain-containing protein, PH domain-containing protein, Na +/H + antiporter subunit E, and pantothenate metabolism flavoprotein (Supplementary Table S3). In addition, we found that the grxC gene was upregulated in both HM and PW (Fig. 8).

## Discussion

The results of this study demonstrate that *S. elongatus* can be effectively utilized in genetic and transcriptomic approaches for the bioremediation of heavy metals and produced water contaminants. The transcriptional responses of *S. elongatus* to these environmental stressors were elucidated through high-throughput RNA sequencing. This discussion places the finding within the context of previous research, to highlight their implications and applications.

Significant differential gene expression was observed in *S. elongatus* exposed to heavy metals (HM) and produced water (PW). Specifically, 11 and 67 genes were upregulated in response to HM and PW, respectively, indicating distinct molecular mechanisms activated under these conditions. The over-expression of the plasma membrane transporter, nitrate ABC transporter permease, suggests its role in bioremediation, aligning with the known functions of ABC transporters in the uptake and transport of heavy metals (Li et al. 2021; Jalmi 2022). Similarly, grxC gene was upregulated in both HM and PW. It encodes glutaredoxin protein, which plays a role in cellular homeostasis by functioning as a thiol-disulfide oxidoreductase to maintain the redox regulation and response to oxidative stress (Chai and Mieyal 2023).

Conversely, the downregulation of 337 and 27 genes under HM and PW conditions, respectively, indicates a complex regulatory network that prioritizes essential survival pathways.

Growth experiments revealed that *S. elongatus* growth declined under produced water conditions but was not negatively impacted by elevated levels of iron. This suggests that the main growth-reducing factor in the produced water is related to the complex mixture of pollutants, including other heavy metals, organic carbon, and other stressors. Additionally, *S. elongatus*’ growth may have been negatively impacted by pathogenic interactions and bacterial competition, as evidenced by the increased bacterial presence in PW samples.

Time-course RNA sequencing revealed details into gene expression dynamics over time. The focus on the 16-h time point was based on the significant number of differentially expressed genes observed. *k*-means clustering analysis identified different expression patterns, with some clusters exhibiting notable alterations in HM and PW conditions. Key stress response mechanisms in *S. elongatus* were highlighted by shared upregulated and downregulated genes between HM and PW treatments. Potential targets for additional research include upregulated genes encoding proteins with the DUF2237, DUF1818, and DUF4160 domains. Downregulated genes indicate potential regions where metabolic processes may be suppressed in response to stress, such as those encoding the circadian clock protein KaiB and the Na^+^/H^+^ antiporter subunit E.

This study demonstrates the importance of transcriptomic analysis in understanding *S. elongatus* stress responses, and findings have significant implications for genetically engineering *S. elongatus* to improve its bioremediation capabilities. The efficiency of bioremediation may be increased by using genetic modification techniques such as CRISPR-Cas9 to target important stress response genes. Given its versatility and potential for bioremediation, the distinct gene expression profiles in response to produced water and heavy metals are evident. Many genes that have been identified in the transcriptome in this study can be targeted in *S. elongatus* by CRIPSR technology to engineer a functional knockout or to modulate the expression of genes (by using CRISPRi; gene repression or CRISPRa: gene activation) (Wang et al. 2023; Boodaghian et al. 2024). CRISPR-Cas9 and CRISPR interference (CRISPRi) have been successfully applied to engineer the cyanobacterium *Synechococcus elongatus* PCC 7942 for various metabolic engineering and gene regulation purposes. For instance, Li et al. (2016) effectively utilized CRISPR-Cas9 to knock-out and knock-in *glgc* and *gltA/ppc* genes, respectively. The gene transformation resulted in improvement of succinate production in *S. elongatus*.

Future investigations should focus on confirming the identified genes and developing genetically modified strains with improved bioremediation abilities. Additionally, investigating how *S. elongatus* interacts with other microbes in wastewater treatment could improve bioremediation methods. Integrating genetic engineering with advanced transcriptomic analysis offers sustainable and effective bioremediation techniques, supporting a clean environment, as well as meeting energy demands.

## Conclusion

In conclusion, this work aimed to explore the transcriptomic response of *S. elongatus* to heavy metals and PW contaminants, using it as a model organism to identify genes potentially involved in bioremediation processes. By applying genetic and transcriptomic approaches, distinct gene expression profiles in response to produced water and heavy metals were identified, including upregulation of genes associated with redox regulation and oxidative stress responses.

These findings demonstrate the importance of transcriptomic analysis in understanding how *S. elongatus* responds to environmental stressors, and they provide a proof of concept for identifying genetic targets relevant to bioremediation. While *S. elongatus* itself may not be the most effective strain for direct PW treatment, its genomic tractability makes it a powerful platform for uncovering key genes and pathways that can be engineered in more robust strains. This work provides potential targets for additional research including upregulated genes encoding proteins that are strongly in relation to improving bioremediation capabilities. Also, future investigations should focus on confirming the identified genes and developing genetically modified strains with improved bioremediation abilities.

## Supplementary Information

Below is the link to the electronic supplementary material.

ESM 1 (PDF 58.8 KB)

ESM 2 (PDF 157 KB)

ESM 3 (PDF 83.3 KB)

ESM 4 (XLSX 29.7 KB)

## Data Availability

No datasets were generated or analysed during the current study.

## Abbreviations

PW: Produced water
HM: Heavy metal
TDS: Total dissolved solids
COD: Chemical oxygen demand
TOC: Total organic carbon
