# Supplementary figures and images for: Gene expression analysis reveals genes related to heavy metals and produced water exposure in Synechococcus elongatus
Source: Int Microbiol. 2025 Sep 22;28(8):2697–708. doi: 10.1007/s10123-025-00715-x (PMC12727749; doi:10.1007/s10123-025-00715-x)

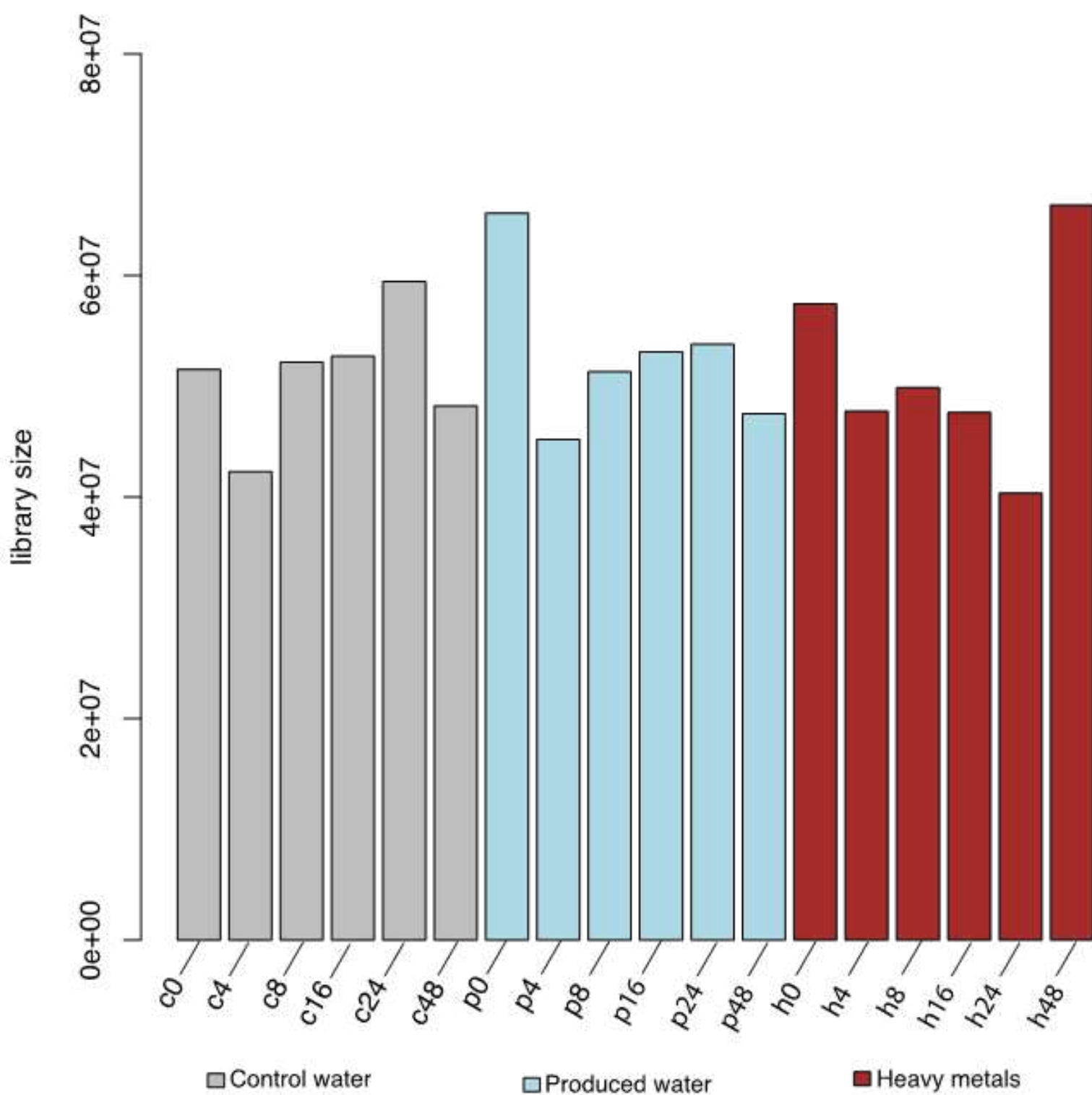

Figure S1 size of row libraries following RNA extraction

Supplement: Supplementary file 1 — (PDF 58.8 KB) [file 10123_2025_715_MOESM1_ESM.pdf]
