# Supplementary material for: Gene expression analysis reveals genes related to heavy metals and produced water exposure in Synechococcus elongatus
Source: Int Microbiol. 2025 Sep 22;28(8):2697–708. doi: 10.1007/s10123-025-00715-x (PMC12727749; doi:10.1007/s10123-025-00715-x)
Supplement: Supplementary file 2 — (PDF 157 KB) [file 10123_2025_715_MOESM2_ESM.pdf]

Table S1 Produced water Characteristics

| Parameter | Units | Results |
|---|---|---|
| Color | - | Light Yellow |
| pH | - | 4.62 |
| Conductivity | uS/cm | 10340 |
| Total Dissolved Solids | mg/L | 6783 |
| Dissolved Oxygen | mg/L | 1.07 |
| Bicarbonate | mg/L | 48.8 |
| Total Alkalinity | mg/L | 40 |
| Salinity | ppt | 8 |
| Chloride | mg/L | 3556 |
| Orthophosphate | mg/L | 2.86 |
| Sulphate ($SO_4$) | mg/L | 46.56 |
| Chemical Oxygen Demand (COD) | mg/L | 55680 |
| Total Organic Carbon (TOC) | mg/L | 14204 |
| **Total Metals** | | |
| Aluminum (Al) | ug/L | 34.64 |
| Barium (Ba) | ug/L | 67.10 |
| Boron (B) | ug/L | 5679 |
| Calcium (Ca) | mg/L | 406.4 |
| Chromium (Cr) | ug/L | 20.84 |
| Copper (Cu) | ug/L | 2.761 |
| Iron (Fe) | ug/L | 1893 |
| Lithium (Li) | ug/L | 3713 |
| Magnesium(Mg) | mg/L | 71.44 |
| Manganese (Mn) | ug/L | 131.0 |
| Nickel (Ni) | ug/L | 1.517 |
| Phosphorus (P) | ug/L | 934 |
| Potassium (K) | mg/L | 134 |
| Silicon (Si) | ug/L | 930 |
| Sodium (Na) | mg/L | 1392 |
| Strontium (Sr) | mg/L | 16.679 |
| Vanadium (V) | ug/L | 2.702 |
| **BTEX** | | |
| Benzene | μg/L | 167604 |
| Toluene | μg/L | 66857 |
| Ethyl benzene | μg/L | 2124 |
| m&p-Xylenes | μg/L | 11658 |
| o-Xylene | μg/L | 5580 |
