# Supplementary material for: Gene expression analysis reveals genes related to heavy metals and produced water exposure in Synechococcus elongatus
Source: Int Microbiol. 2025 Sep 22;28(8):2697–708. doi: 10.1007/s10123-025-00715-x (PMC12727749; doi:10.1007/s10123-025-00715-x)
Supplement: Supplementary file 3 — (PDF 83.3 KB) [file 10123_2025_715_MOESM3_ESM.pdf]

Table S2 List of genes that are dysregulated considering absolute logFC 1 in HM and PW at different time points

Time (hour)	PW vs. Control (Up-regulated)	PW vs. Control (Down-regulated)	HM vs. Control (Up-regulated)	HM vs. Control (Down-regulated)
4	520	549	235	141
8	630	664	212	247
16	719	671	699	766
24	694	757	477	463
48	518	694	342	295
